# Neuroprotective effect of extracellular vesicles of human glial progenitor cells mediated by activation of PI3K‐AKT signaling pathway

**DOI:** 10.1002/alz70859_099689

**Published:** 2025-12-25

**Authors:** Margarita Shedenkova, Diana Salikhova, Anastasia Nekrasova, Zanda Bakaeva, Dmitry Goldshtein

**Affiliations:** ^1^ RUDN University, Moscow, Moscow Russian Federation; ^2^ National Medical Research Center for Children’s Health, Moscow, Moscow Russian Federation; ^3^ Research Centre for Medical Genetics, Moscow, Moscow Russian Federation

## Abstract

**Background:**

Extracellular vesicles (EVs) have recently entered the group of modern neurotherapeutic agents for Alzheimer disease treatment. Glutamate excitotoxicity remains the most common and damaging consequence of this neurodegeneration. In this work, we investigate the neuroprotective mechanisms of a new type of EVs derived from human glial progenitor cells (hGPCs) using proteomic and inhibitor assays in the glutamate excitotoxicity model.

**Method:**

EVs were obtained by ultracentrifugation from conditioned medium of hGPCs. Glutamate excitotoxicity model was established using rat pups (P0) primary cultures of cortical neurons. To perform proteomic analysis of EV‐GPCs was used an Ultimate 3000 Nano LC System chromatography system interfaced with a Q Exactive HF mass spectrometer. The proteins peak list was generated and analyzed by MASCOT using the UniProtKB database. Proteins systematization and categorization was performed using the String 8.0 database. To confirm the activation of founded PI3K‐Akt signaling pathway a selective inhibitor AS605240 of PI3Kγ subunit was used.

**Result:**

In the model of glutamate excitotoxicity, a 37.5% decrease in the viable neurons number was observed with the addition of glutamate compared to the control group. The presence of EV‐hGPCs (3 μg/mL) significantly increased survival rates by 20%, whereas addition of EV‐GPCs (10 μg/mL) increased survivability to control values. According to the bioinformatic analysis, the most represented protein groups of the signaling pathways (KEGG database) in EVs was the "PI3K‐Akt signaling pathway" (51 proteins). To confirm that EVs can activate this pathway in cells, inhibitor assay was performed using AS605240 (1 μg/mL). Its addition completely offset the drug’s neuroprotective effect.

**Conclusion:**

The obtained results indicate that EV‐hGPCs have neuroprotective effect on the glutamate excitotoxicity model. This is due to the proteins they contain that activate the PI3K‐Akt signaling pathway.

**Funding**: The work was financially supported by RSF grant № 23‐15‐00362 “Study of mechanisms of the therapeutic action of extracellular vesicles derived from human glial progenitor cells on the model of Alzheimer's disease”

